# Strengthening Global Health Security Capacity — Vietnam Demonstration Project, 2013

**Published:** 2014-01-31

**Authors:** Tran Dac Phu, Vu Ngoc Long, Nguyen Tran Hien, Phan Trong Lan, Wayne Lowe, Michelle S. McConnell, Michael F. Iademarco, Jeffrey M. Partridge, James C. Kile, Trang Do, Patrick J. Nadol, Hien Bui, Diep Vu, Kyle Bond, David B. Nelson, Lauren Anderson, Kenneth V. Hunt, Nicole Smith, Paul Giannone, John Klena, Denise Beauvais, Kristin Becknell, Jordan W. Tappero, Scott F. Dowell, Peter Rzeszotarski, May Chu, Carl Kinkade

**Affiliations:** 1 Vietnam Ministry of Health; 2 National Institute of Hygiene and Epidemiology; 3 Pasteur Institute - Ho Chi Minh City; 4 Defense Threat Reduction Agency, US Department of Defense; 5 Division of Global HIV/AIDS, Center for Global Health, CDC; 6 Division of TB Elimination, National Center for HIV/AIDS, Viral Hepatitis, STD, and TB Prevention, CDC; 7 Influenza Division, National Center for Immunization and Respiratory Disease, CDC; 8 Center for Global Health, CDC; 9 Office of Public Health Preparedness and Response, CDC; 10 Center for Surveillance, Epidemiology, and Laboratory Services, CDC

Over the past decade, Vietnam has successfully responded to global health security (GHS) challenges, including domestic elimination of severe acute respiratory syndrome (SARS) and rapid public health responses to human infections with influenza A(H5N1) virus (1). However, new threats such as Middle East respiratory syndrome coronavirus (MERS-CoV) and influenza A(H7N9) present continued challenges, reinforcing the need to improve the global capacity to prevent, detect, and respond to public health threats. In June 2012, Vietnam, along with many other nations, obtained a 2-year extension for meeting core surveillance and response requirements of the 2005 International Health Regulations (IHR) (2,3). During March–September 2013, CDC and the Vietnamese Ministry of Health (MoH) collaborated on a GHS demonstration project to improve public health emergency detection and response capacity. The project aimed to demonstrate, in a short period, that enhancements to Vietnam’s health system in surveillance and early detection of and response to diseases and outbreaks could contribute to meeting the IHR core capacities, consistent with the Asia Pacific Strategy for Emerging Diseases (4). Work focused on enhancements to three interrelated priority areas and included achievements in 1) establishing an emergency operations center (EOC) at the General Department of Preventive Medicine with training of personnel for public health emergency management; 2) improving the nationwide laboratory system, including enhanced testing capability for several priority pathogens (i.e., those in Vietnam most likely to contribute to public health emergencies of international concern); and 3) creating an emergency response information systems platform, including a demonstration of real-time reporting capability. Lessons learned included awareness that integrated functions within the health system for GHS require careful planning, stakeholder buy-in, and intradepartmental and interdepartmental coordination and communication.

To ensure that project enhancements were built on existing MoH systems and structures, initial planning was coordinated by the General Department of Preventive Medicine and focused on identifying existing capacity and needs. MoH has a functioning health response system, including organizational and physical infrastructure. Formal documents delineate authorities.* An electronic communicable disease reporting system aggregates data from 48 provinces with planned expansion to all 63 by 2014. This system collects data regularly on Vietnam’s 28 reportable conditions, according to standard case definitions. Sentinel systems are set up for certain infectious diseases (e.g., HIV; influenza; cholera; plague; and enterovirus 71, the causative agent for hand, foot, and mouth disease in Vietnam associated with severe neurologic disease). There are four regional public health institutes that are responsible for epidemiologic surveillance, response, and laboratory confirmation for priority pathogens. The World Health Organization (WHO) and CDC-supported National Influenza Center laboratories at the National Institute of Hygiene and Epidemiology (NIHE) in Hanoi and at the Pasteur Institute–Ho Chi Minh City (PI-HCMC) are responsible for detection of seasonal and avian influenza viruses, and the institutes’ virology departments are responsible for detection and response to emerging pathogens such as MERS-CoV and established priority pathogens such as dengue and hand, foot, and mouth disease (Figure). Infectious disease rapid response teams are established at central to district levels, and a 2-year Field Epidemiology Training Program,† established in 2009, graduated its first cohort in 2011.

## GHS Demonstration Project

In March 2013, a GHS team was formed, and the project received strong support from MoH leadership with official approval in April. MoH issued Decision 1424 on May 2 to establish an EOC office comprising MoH departments, regional public health institutes, and relevant international agencies, including WHO, the United Nations’ Food and Agriculture Organization, and CDC. In-country CDC staff members from the Influenza Division and the Division of Global HIV/AIDS assumed leadership roles for the provision of technical assistance for emergency operations, laboratory systems, and information systems. The team was augmented from CDC headquarters, including experts from seven other divisions. Activities to enhance laboratory and information systems built on foundations laid by CDC programs in Vietnam starting in 2000. Following stakeholder discussions, including with the U.S. Agency for International Development, U.S. Department of Defense, and WHO, a precise activity plan was developed, detailing resources for staffing, technical support, and procurement of supplies and equipment.

## Emergency Operations Center

A core EOC team was established at MoH, and CDC experts assisted to develop an emergency operations handbook with standard operating procedures and forms tailored to meet existing Vietnamese policies and regulations. The operations handbook contained internationally recognized functions and procedures for managing, responding to, and reporting disease outbreaks and other emergencies. Emergency operations training of MoH personnel was provided in-country (30 participants), at the EOC at CDC headquarters (two groups of three participants each), and at the EOC at the WHO Western Pacific Regional Office (three participants). Different international operations center models were reviewed, and plans were developed consistent with options at MoH for renovation of existing office space, relocation of existing staff, and installation of necessary equipment.

## Laboratory Systems

Work was focused at two of the four regional public health institutes, NIHE and PI-HCMC. Laboratory assessments of the influenza and enterovirus laboratories were conducted, and equipment and supplies required for application of the new testing platform were determined (5). Staff members from these two laboratories were trained in the WHO- and CDC-approved real-time reverse transcription–polymerase chain reaction (rRT-PCR) assay for influenza A(H7N9) detection, and in new testing platforms using rRT-PCR for detection of enterovirus 71 (EV71), and in multiplex PCR for detection of seven respiratory pathogens.§ Quality management systems were reviewed, including the National Laboratory Strategic Plan and Strengthening Laboratory Management Toward Accreditation and international level laboratory accreditation platform (ISO15189), all supported by the President’s Emergency Plan for AIDS Relief (PEPFAR) (6). In addition to the work at NIHE and PI-HCMC, mapping of the national laboratory system was begun to allow strengthening of the network for sample shipment, testing, reporting, and referral (7).

## Information Systems

To enhance biosurveillance and information systems using the backbone of the MoH’s electronic communicable disease surveillance system, CDC’s Epi Info tools were developed in Vietnamese to enhance analysis and real-time reporting of disease surveillance data for MoH decision makers. The use of Epi Info as an accessible, flexible, and comprehensive data collection, management, and analysis tool for investigations was demonstrated to MoH staff. A plan was developed to incorporate Epi Info into the toolkit used by MoH rapid response teams responsible for investigating outbreaks.

## Drills

At the project’s September 2013 conclusion, a series of functional interrelated drills were conducted to 1) verify accuracy of laboratory testing by matching reported results to known but blinded panels containing specific pathogens; 2) assess performance by measuring turnaround times from sample receipt to results reporting; 3) provide a training opportunity for MoH EOC staff members and subcommittees of the National Steering Committee to practice EOC functions in a controlled scenario; and 4) confirm data transmitted across systems received at each designated point in the communications network. Two 3-day laboratory drills were conducted separately at NIHE and PI-HCMC. Mock drill panels for rRT-PCR were supplied by CDC and Oxford University Clinical Research Unit.¶ Both laboratories accurately identified all pathogens in their panels using the new algorithms within the required 48-hour timeframe, in accordance with IHR reporting requirements.

The 2-day emergency operations drill, led by MoH and assisted by CDC and Defense Threat Reduction Agency experts, included participation and coordination by multiple MoH groups, the Ministry of Agriculture and Rural Development, and international partners. Strengths identified from the drill included effective communication and problem-solving; a notable outcome was the creation and review of an Incident Action Plan.

### Editorial Note

By leveraging existing U.S. government and Vietnamese investments and building on existing platforms, enhancements to GHS were made within a short period, allowing for accurate and timely testing of emerging pathogens and increased ability to manage a public health emergency through an EOC. Project enhancements included 1) training and infrastructure, 2) support for laboratories for improved detection of priority pathogens using rRT-PCR and a multiplex PCR platform, 3) development of an operations handbook with standard procedures and forms and training materials for improved management at the existing MoH EOC, and 4) adaptation of Epi Info tools, allowing enhanced analysis and reporting of data from existing communicable disease surveillance systems.

What is already known on this topic?

New threats such as Middle East respiratory syndrome coronavirus and influenza A(H7N9) present continued challenges and highlight the need for countries to improve their capacity to prevent, detect, and respond to public health threats. In June 2012, Vietnam, along with many other nations, obtained a 2-year extension for meeting core surveillance and response requirements of the 2005 International Health Regulations.

What is added by this report?

During March–September 2013, CDC collaborated with the Vietnamese Ministry of Health on a project to demonstrate that enhancements could be made in a short period to the capacity for surveillance and early detection of and response to disease outbreaks in Vietnam. Achievements included enhanced laboratory testing capability for several priority pathogens, established emergency operations functions, and demonstration of the need and capability for information systems to enhance public health emergency reporting.

What are the implications for public health practice?

This is a successful model for other nations with similar health systems to increase prevention, detection, and response capability to public health threats. Careful planning, stakeholder buy-in, and intradepartmental and interdepartmental coordination and communication are required.

Lessons learned included the importance of rapid data transmission and sharing, the need to promote application of information technology in disease surveillance and outbreak response, and the need for intra-agency and interagency coordination and collaboration. Application of technology in disease surveillance reduces the time for data collection, reporting, analysis, and sharing, thereby enhancing early detection and rapid response to diseases and outbreaks. In addition, installation of and training on new testing platforms allowed for harmonization of protocols for selected pathogens across the regional institutes’ laboratories. Review of the National Laboratory Strategic Plan developed under PEPFAR confirmed it to be an important framework with relevance to public health laboratories and highlighted the importance to GHS of quality management systems (8). As a result of the project, CDC and MoH engaged in a substantive dialog about a broader set of pathogens for early detection and rapid response. EOC, with the enhancements of necessary procedures and equipment, will serve as a working body to assist the National Steering Committee on Emerging Disease Control and Prevention. The emergency operations drill and training, following the new operations handbook, built MoH capacity to design and run their own exercises, moving beyond externally led table top exercises.

Challenges identified by MoH included limited resources (staffing, infrastructure, funding, and reagents) for GHS activities, a limited understanding of GHS by MoH agencies and other stakeholders, varied coordination and collaboration between different agencies and ministries, a lack of harmonization of laboratory diagnostics and data management, and limited data sharing and application of information technology in surveillance systems. International models and guidelines need to be adapted to the existing polices, structures, and systems to be integrated and sustainable. Despite these challenges, Vietnam and the United States collaborated to make discernible improvements in existing GHS capabilities in a short period, moving Vietnam closer to IHR compliance with all core capacities.** This multisectorial approach to capacity building for public health emergencies has the potential to serve as a model for similar collaborations elsewhere.

## Figures and Tables

**FIGURE:**
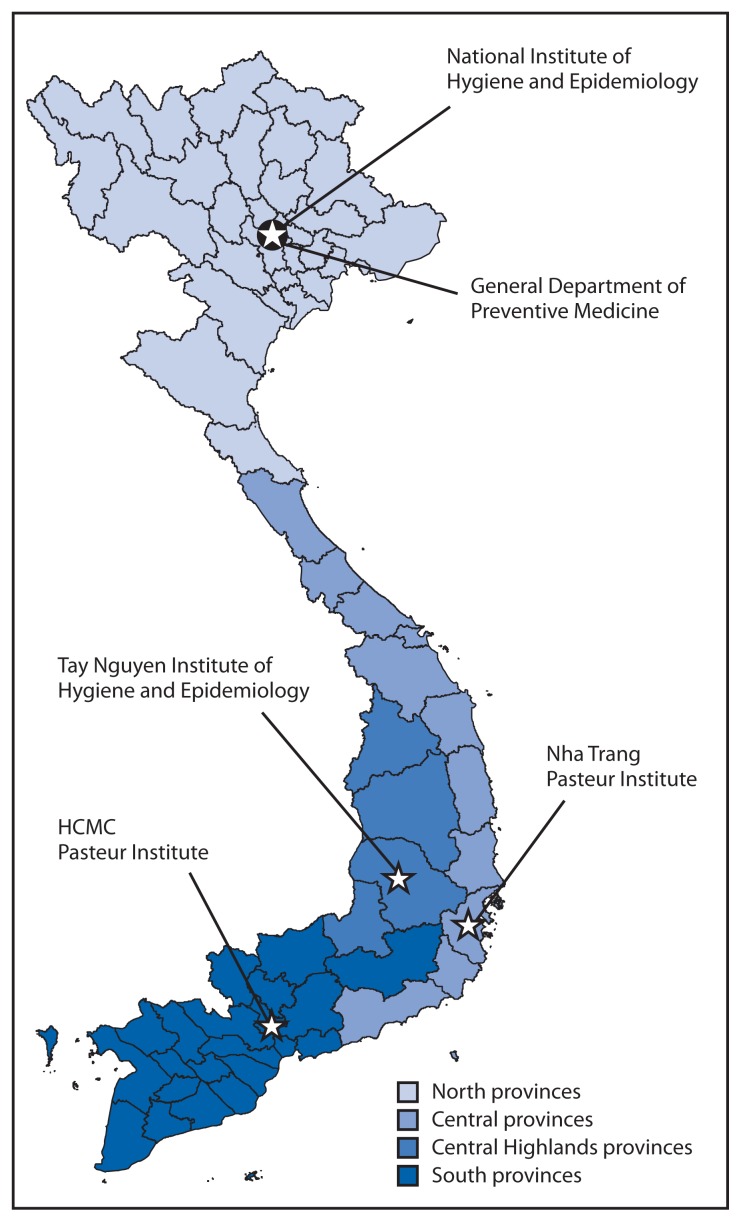
The four regional public health institutes^*^ and their provinces of responsibility for epidemiologic surveillance, response, and laboratory confirmation and the General Department of Preventive Medicine^†^ — Global Health Security demonstration project, Vietnam, 2011 **Abbreviation:** HCMC = Ho Chi Minh City. ^*^The National Institute of Hygiene and Epidemiology, Tay Nguyen Institute of Hygiene and Epidemiology, Nha Trang Pasteur Institute, and the Ho Chi Minh City Pasteur Institute. ^†^The focal point in the Vietnam Ministry of Health for the Global Health Security demonstration project.

## Footnotes

* A National Steering Committee for the Prevention and Control of Dangerous and Emerging Diseases exists with five subcommittees devoted to surveillance, treatment, logistics, communication, and international cooperation.

† CDC works with foreign MoHs to support Field Epidemiology Training Programs modeled after CDC’s Epidemic Intelligence Service. Additional information available at http://www.cdc.gov/globalhealth/fetp.

§ Respiratory syncytial virus; human metapneumovirus; parainfluenza viruses 1, 2 and 3; adenovirus; and MERS-CoV.

¶ Samples for NIHE consisted of inactivated seasonal influenza A, influenza B, avian influenza A(H7N9), MERS-CoV, and negative samples. Samples for PI-HCMC consisted of EV71 viruses (at different concentrations), avian influenza A(H5N1), and negative samples.

** The IHR core capacities are 1) national legislation, policy, and financing, 2) coordination and national focal point communications, 3) surveillance, 4) response, 5) preparedness, 6) risk communication, 7) human resources, and 8) laboratory. Additional information available at http://www.who.int/ihr/Processes_of_IHR_Monitoring_framework_and_Indicators.pdf.
